# Non-mosaic partial duplication 12p in a patient with dysmorphic characteristics and developmental delay

**DOI:** 10.1590/1678-4685-GMB-2018-0285

**Published:** 2020-02-10

**Authors:** Jakeline Santos Oliveira, Tatiana Mozer Joaquim, Rosana Aparecida Bicudo da Silva, Deise Helena de Souza, Lúcia Regina Martelli, Danilo Moretti-Ferreira

**Affiliations:** 1 Universidade Estadual Paulista “Júlio de Mesquita Filho” (UNESP), Instituto de Biociências, Departamento de Ciências Químicas e Biológicas, Botucatu, SP, Brazil.; 2 Universidade de São Paulo, Faculdade de Medicina de Ribeirão Preto, Departamento de Genética, Ribeirão Preto, SP, Brazil.

**Keywords:** Duplication 12p, *array*-CGH, facial dysmorphism

## Abstract

Duplication of the short arm of chromosome 12 is a rare chromosomal abnormality that may arise *de novo* or result from malsegregation of a balanced parental translocation. This study comprises the clinical description, cytogenetic and cytogenomic analyses and genotype-phenotype correlation in a patient with facial dysmorphism, developmental delay and intellectual impairment caused by non-mosaic partial duplication and a paracentric inversion 12p. The patient’s GTG-banded karyotype was 46,XX,invdup(12)(pter → p13.32::p11.1 → p13.31::p13.31 → qter). A genetic gain of approximately 28 Mb was detected in the chromosomal region arr[GRCh37]12p13.31-p11.1(6914072_34756209)x3. The chromosomal alteration seen in our patient is described as “pure” partial duplication 12p. In most cases, duplication 12p phenotype is characterized by dysmorphic features, multiple congenital anomalies and intellectual disability. A small number of cases in literature have described genes associated with neurodevelopmental disease, such as *ING4*, *CHD4*, *MFAP5, GRIN2B*, *SOX5*, *SCN8A* and *PIANP*. In our patient the duplication 12p was *de novo*. This study should contribute to the genotype-phenotype correlation in partial duplication 12p cases.

Duplication of the short arm of chromosome 12, first described by Uchida and Lin (1973), is a rare chromosomal abnormality with an estimated incidence of 1/50,000 live births (Stengel-Rutkowski *et al.*, 1981). According to (Allen *et al.*, 1996) cases of duplication 12p can be divided into five categories based on the extent of the region duplicated and whether other chromosomal aneusomies are present. Category I is designated as a partial “pure” trisomy of 12p with a duplication point distal to 12p11 and not involving any other chromosome. Category II includes cases with trisomies 12p in association with cell-line mosaicism. Category III includes cases with complete and “pure” 12p trisomy with an additional trisomy or monosomy of the short arm of an acrocentric chromosome. Category IV and V involves complete trisomy 12p with monosomy or trisomy of non-acrocentric chromosomes other than 12p, or trisomic involvement of 12q, respectively. The duplication of 12p is defined as “complete” when there is duplication region of 12p11 or 12p12-12pter. The duplication of 12p is considered “pure” as having no other aneusomy or additional aneusomies of only the pter regions of non-acrocentric chromosomes, and not involving mosaicism (Allen *et al.*, 1996).

By these criteria, (Liang *et al.*, 2006) compared 23 patients with “pure” trisomy 12p, subdividing the cases into four subgroups (A, B, C and D) to refine the karyotype-phenotype correlation based on the extent of 12p-duplicated region involved. Subgroup A is complete 12p trisomy, subgroup is B terminal trisomy 12p, subgroup is C distal trisomy 12p and subgroup is D proximal trisomy 12p.

Approximately 50 cases of duplications 12p have been described in the medical literature to date consisting of small studies and case reports, that are limited to descriptions of the clinical syndrome at birth or in early infancy. Little is known about the life expectancy of these children, beyond infancy and most of the diagnostic criteria are based on descriptions of infants and adolescents (Segel *et al.*, 2006; Inage *et al.*, 2010; Liu *et al.*, 2012; Poirsier *et al.*, 2014; Mekkawy *et al.*, 2016). The clinical signs most commonly associated with duplication 12p are increased weight at birth, hypotonia, craniofacial anomalies such as turricephaly, macrocephaly, round face, full cheeks, frontal bossing, wide nasal bridge, short nose, anteverted nares, long philtrum, thin upper lip, short neck, dysmorphic ears, intellectual impairment and moderate to severe psychomotor delay (Hung *et al.*, 2012; Poirsier *et al.*, 2014).

Some dysmorphic features such as sparse hair and eyebrows, hypertelorism, wide and depressed nasal bridge, short nose with wide and anteverted nares, up-slanting palpebral fissures, epicanthic folds, full cheeks, long philtrum, and short neck seen in patients with 12p duplication overlap with patients with Pallister-Killian syndrome (PKS) (OMIM: 601803) (Inage *et al.*, 2010). The PKS is typically caused by the presence of a supernumerary isochromosome composed of the short arms of chromosome 12, generating tetrasomy 12p, which is often present in a tissue limited mosaic state (Peltomäki *et al.*, 1987). However, some characteristics such as pigmentary skin differences, diaphragmatic hernia, congenital heart defects, and other systemic abnormalities are present only in patients with PKS (Izumi *et al.*, 2012).

In most of the reported cases, duplication 12p resulted from malsegregation of a balanced parental translocation (Segel *et al.*, 2006). Duplication 12p may also arise *de novo* from misalignment of low copy repeats (LCRs) through non-allelic homologous recombination (NAHR) (De Gregori *et al.*, 2005).

The present study included the clinical description, cytogenetic and cytogenomic analyses, as well as genotype-phenotype correlation in a patient with facial dysmorphism, developmental delay and intellectual impairment caused by non-mosaic partial duplication 12p, *de novo*.

This 15-year-old girl, the only child of young and healthy non-consanguineous parents, was born at 40 weeks after uneventful pregnancy and delivery. Her birth weight was 2.535 kg (p 2.5), and length 48 cm (p>2.5). She presented jaundice at birth, and, therefore, received phototherapy for five days. At one year, the patient showed having seizures with recurrence. In the first genetics clinical evaluation, at 2,1 yo, height 86 cm (p<3.95), weight 12.5 kg (p<3.58) and OCP 47.5 cm (percentile < 2). Showed a broad forehead, flat face, narrow eye slits, microblepharon, anteverted nostrils, low nasal root with a broad nasal base, smooth nasolabial philtrum, tent-shaped upper lip, (Figure 1) scoliosis, single simian crease in the left hand, bilateral flat foot, right *genu valgum*, developmental delay and intellectual disability. In the follow up at 13 yo, information was that she could walk and talk when she was 3 yo. At 6 yo, she underwent surgical correction of *genu valgum* and showed short lower limbs. She developed thelarche at 10 yo, and pubarche at 11 yo. At that time, her cholesterol was 600 mg/dL, and she had hypothyroidism, good comprehension but unable to read or write.

**Figure 1:**
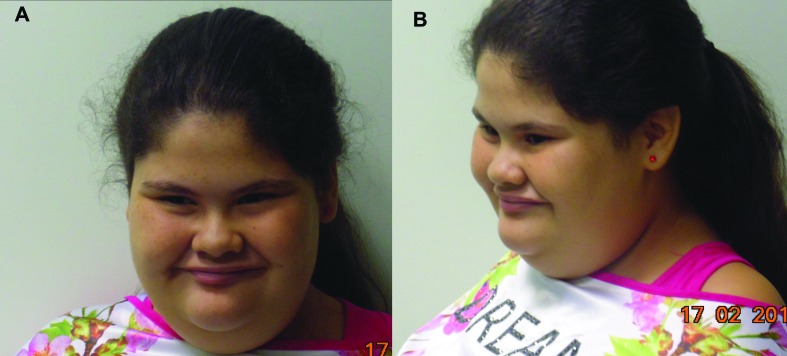
Patient at 13 years. (A) Frontal view. (B) Side view. Note the frontal humps, flat cheeks, round face, full cheeks, short nose, low nasal bridge, thin upper lip and short neck.

Chromosomal analysis was carried out on temporary peripheral lymphocyte cultures obtained from the patient and her parents as described by Moorhead and Furman (1960), with modifications. GTG banding (550 bands) was performed as described by Seabright (1971) and high-resolution banding according to Yunis (1976), both with modifications. The patient’s GTG-banded karyotype was 46,XX,invdup(12)(pter → p13.32::p11.1 → p13.31::p13.31 → qter) while the karyotype of both parents were normal. FISH analysis, performed according to the instructions of the manufacturer, using a WCP chromosome 12 probe (Cytocell Aquarius^TM^ Oxford Gene Technology), revealed that one of the homologous chromosomes differed in size (Figure 2).

**Figure 2:**
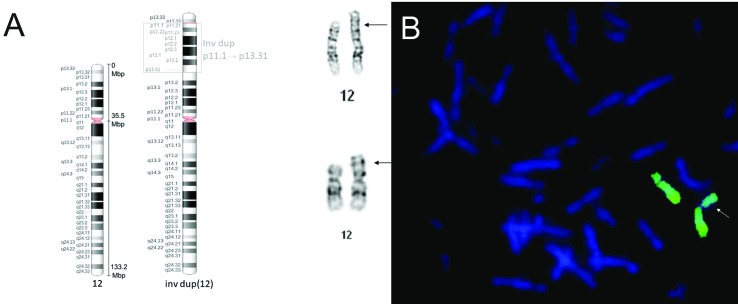
Chromosome 12 karyotyping and FISH. (A) GTG-banded karyotype of patient showing the normal and duplication and inversion paracentric 12p. (B) FISH metaphase using WCP probe for chromosome 12 of *CYTOCELL* AQUARIUS® Oxford Gene Technology indicating a difference in size in one of the patient’s homologous chromosome, the arrow indicates the duplication 12 p.

For cytogenomic analysis, DNA was extracted from 200 μL of the patient’s whole blood using the MasterPure Complete DNA and RNA Purification Kit (Epicentre, US) according to the manufacturer’s instructions. Array-CGH was performed using the GenetiSure^TM^ CGH + SNP 4x180k platform (Agilent). The data were analyzed using Nexus Copy Number software (Biodiscovery v. 8.0). Our analyses were based on the reference genome GRCh37/hg19 and online databases such as UCSC Genome Browser (GRCh37/hg19), Database of Genomic Variants (DGV), Database of Chromosomal Imbalance and Phenotype in Humans Using Ensembl Resources (DECIPHER), Online Mendelian Inheritance in Man (OMIM) and PubMed. The array-CGH analysis showed a large genetic gain of approximately 27.842,138 base pairs (~ 28 Mb) arr[GRCh37]12p13.31-p11.1 (6914072_34756209)x3, including 282 genes (Figure 3).

**Figure 3:**
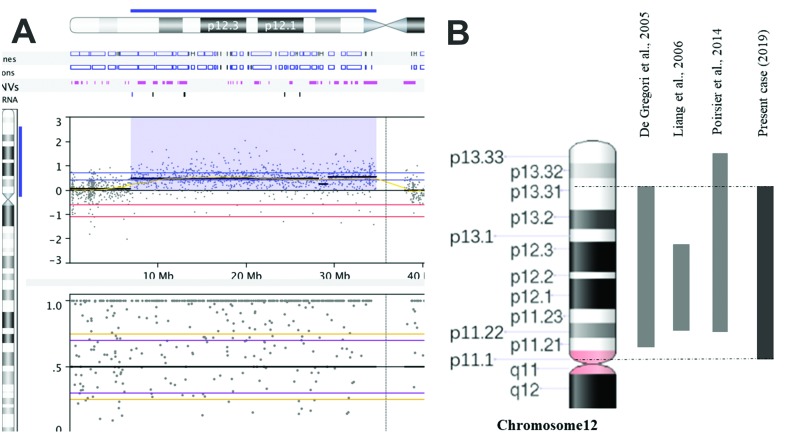
Chromosome 12 characterization. (A) Array-CGH of chromosome 12, the color blue indicates the duplicated chromosomal region 12p13.31 → p11.1 of ~28 Mb in size. (B) Duplication 12p patients reported in the literature with chromosome region similar to that of the present case.

The first patient with a duplication 12p was clinically diagnosed initially as having Down syndrome, due to the similarity of flat face and clinical features such as epicanthus, broad nasal bridge, bilateral simian creases, abnormal dermal patterns and intellectual disability. However, chromosomal analysis by quinacrine dihydrochloride was revealed a trisomy 12p and monosomy of the distal region of chromosome 8 (Uchida and Lin, 1973).

Since then, several researchers aimed at correlating the karyotype-genotype-phenotype with duplication 12p (Inage *et al.*, 2010). However, duplication 12p is a chromosomal alteration with little recurrence of cases in the literature. In addition, there is a great phenotypic variability due to the different sizes and chromosome breaks of the 12p duplication (Eid *et al.*, 2014).

De Gregori *et al.* (2005) describe the characterization of an interstitial duplication of 12p, dup (12) (p11.21 → p13.31), by array-CGH and FISH in a patient with mental retardation and dysmorphic features. They hypothesized that the duplication 12p could have been generated by homology of three blocks of low copy repeats (LCRs) flanking the duplication region. They suggested that misalignment of these LCRs could have mediated the recurrent rearrangement type *de novo*.

The duplicated chromosomal region in our patient is similar to that described by De Gregori *et al.* (2005), and in keeping with the LCRs model, the origin of the duplication in our patient is *de novo*. However, in our patient the orientation of the duplicated chromosomal segment is inverted.

The karyotype-phenotype correlation in our patient allows us to characterize the extent of the duplicate chromosome fragment into Category I and, depending on the extent of duplication, in the subgroup of group D (Allen *et al.*, 1996; Liang *et al.*, 2006). Category I is said to be the most benign group in trisomy 12p, in addition to points of 12p distal to 12p11 (Allen *et al.*, 1996).

Among the subgroups of “pure” partial trisomy, the clinical features, such as broad ears and deformities in the feet were mapped to a segment of 5 Mb, the 12p13.1-p12.3 segment, because they were present in subgroup A and C patients, but not in subgroups B and D patients (Allen *et al.*, 1996; Rauch *et al.*, 1996; Zumkeller *et al.*, 2004; Liang *et al.*, 2006). In our patient these clinical features are not present.

Epicanthal folds, ear anomalies, short neck, and round face/prominent cheeks were observed in groups A, B, and C patients, but not in group D patients, suggesting that these features may be associated with a segment telomeric to 12p12.3 (Tekin *et al.*, 2001; Tsai *et al.*, 2005; Liang *et al.*, 2006). However in our patient, round face/prominent cheeks and short neck were described, in addition to the 12p12 region duplication. The common phenotype is in most cases independent of the category, being facial dysmorphism, and developmental delay (Allen *et al.*, 1996; Rauch *et al.*, 1996).

Compared with cases described in the literature with “pure” partial duplication 12p similar to the duplication region of our patient, the clinical description included a round face, full checks, proeminent forehead/frontal, short nose, wide/depressed nasal bridge, anteverted nostril, long/deep philtrum, large downward facing mouth, and inverted lower lip (Table 1) are present in most cases of Category I and subgroup D (De Gregori *et al.*, 2005; Liang *et al.*, 2006; Poirsier *et al.*, 2014).

**Table 1:** Comparison of the clinical features of patients with “pure” duplication 12p subgroup D.

Clinical features	De Gregori *et al*., 2005	Liang *et al*., 2006	Poirsier *et al*., 2014	Present case
	p11.21 → p13.31	inv dup(12) p12.3 → p11.22	Patient 1 p13.33 → p11.21	inv dup(12) p13.31 → p11.1
Round face	+	-	+	+
Full cheeks	NR	+	+	+
Prominent forehead/Frontal	+	+	+	+
Hypertelorism	NR	NR	+	-
Epicanthus	NR	-	+	-
Occipital plane	NR	NR	NR	-
Short nose	+	+	+	+
Wide/Depressed nasal bridge	+	+	+	+
Anteverted nostril	+	+	+	+
Long/deep philtrum	+	-	+	+
Micrognathia	NR	NR	+	+
Big mouth facing down	+	+	+	+
Thin upper lip	NR	NR	+	+
Inverted lower lip	+	+	+	+
Low-Eyed ears	+	+	+	-
Dysmorphic ears	NR	+	+	-
Short neck	NR	-	+	+
Hypotonia	NR	-	+	+
Seizure	NR	NR	-	+
Developmental delay	+	+	+	+

+, feature present; -, negative feature; nr, not reported/not determined.

The clinical findings, such as hypotonia, high forehead, prominent cheeks, flat face, large philtrum, short nose with anteverted nostril, broad everted lower lip, and short neck are in agreement with the partial duplication 12p in our patient. Although our patient did not present increased birth weight, she is currently overweight (Rauch *et al.*, 1996).

The duplicated 12p13.1 region present in patients previously described in the literature contributes to the hypothesis that this region contains genes that are sensitive to gene dosage and that this region could be responsible for facial dysmorphism (Rauch *et al.*, 1996; Tsai *et al.*, 2005). This region is also altered in cases of PKS with overlapping facial features for 12p duplication/PKS (Izumi *et al.*, 2012). The diagnosis of PKS requires the identification of mosaic isochromosome 12p by conventional karyotyping and FISH (Hung *et al.*, 2012). Our patient showed clinical features consistent with a duplication 12p, but since no isochromosome 12p was detected by karyotyping and FISH, the patient was diagnosed with non-mosaic partial duplication 12p.

Izumi *et al.* (2012) hypothesized that duplication of the genes located within 12p13.31 might be sufficient to result in the core phenotype of 12p duplication/PKS. According to these authors, *ING4*, *CHD4* and *MFAP5*, represent strong candidate genes, given their important roles in cell proliferation and differentiation. Moreover, alterations in these genes have been associated with neurological disorders.

According to Segel *et al.* (2006), genes that are important for early morphogenesis are affected by trisomy, while genes that are important for fetal and placental growth are not. However, genes responsible for brain development (functional and possibly structural) continue to be affected throughout pregnancy, resulting in poor brain development, and trisomy 12p influences the early developmental milestones, as well as cognitive and neurological function. Patients with trisomy 12p chromosome tend to have seizures at seven or eight years of age. In our patient, the seizures began in the first years of life, becoming recurrent. She also has speech and motor delay, and intellectual disability.

Currently, the use of array-CGH, is aimed at the elucidation of genetic variants, such as Copy Number Variants (CNVs) that can overlap genes and consequently alter the phenotype. The study of (Coe *et al.*, 2014) correlated CNVs associated with many neurocognitive disorders in individuals with developmental delay. Among the mapped gain/duplication type CNVs, these overlap the *GRIN2B*, *SOX5*, *SCN8A* and *PIANP* genes in the 12p12.1 and 12p13.3 regions. These genes act in the central nervous system and craniofacial development and are potential candidates for neurological changes in patients with 12p duplication (Poirsier *et al.*, 2014).

Most reports of cases of duplication 12p describe the clinical characteristics of the patients and conventional cytogenetics analysis. Only for six cases of duplication 12p, an array-CGH analysis was performed to refine the chromosomal break points and describe the genes inserted in the duplicated chromosomal region, thus improving the genotype-phenotype correlation (De Gregori *et al.*, 2005; Hung *et al.*, 2012; Izumi *et al.*, 2012; Liu *et al.*, 2012; Poirsier *et al.*, 2014; Mekkawy *et al.*, 2016).

In conclusion, we describe a case of a rare chromosomal imbalance leading to partial duplication 12p and inversion of *de novo* origin that could have been generated as a result of the misalignment of LCR blocks. The phenotype-karyotype correlation showed similarities to previously reported cases of partial duplications 12p. Few cases in literature have described genes associated with neurodevelopmental disease, such as *ING4*, *CHD4*, *MFAP5, GRIN2B*, *SOX5*, *SCN8A* and *PIANP*. Our results contribute to the hypothesis that the 12p13.3 region is responsible for most of the dysmorphic features of duplication 12p.
